# Increased Attack Rates and Decreased Incubation Periods in Raccoons with Chronic Wasting Disease Passaged through Meadow Voles

**DOI:** 10.3201/eid2804.210271

**Published:** 2022-04

**Authors:** S. Jo Moore, Christina M. Carlson, Jay R. Schneider, Christopher J. Johnson, Justin J. Greenlee

**Affiliations:** US Department of Agriculture, Ames, Iowa, USA (S.J. Moore, J.J. Greenlee);; US Geological Survey National Wildlife Health Center, Madison, Wisconsin, USA (C.M. Carlson, J.R. Schneider, C.J. Johnson).

**Keywords:** chronic wasting disease, wild animals, central nervous system, North America, prions and related diseases, veterinary medicine, raccoons, voles

## Abstract

Chronic wasting disease (CWD) is a naturally-occurring neurodegenerative disease of cervids. Raccoons (*Procyon lotor*) and meadow voles (*Microtus pennsylvanicus*) have previously been shown to be susceptible to the CWD agent. To investigate the potential for transmission of the agent of CWD from white-tailed deer to voles and subsequently to raccoons, we intracranially inoculated raccoons with brain homogenate from a CWD-affected white-tailed deer (CWD^Wtd^) or derivatives of this isolate after it had been passaged through voles 1 or 5 times. We found that passage of the CWD^Wtd^ isolate through voles led to a change in the biologic behavior of the CWD agent, including increased attack rates and decreased incubation periods in raccoons. A better understanding of the dynamics of cross-species transmission of CWD prions can provide insights into how these infectious proteins evolve in new hosts.

Transmissible spongiform encephalopathies, or prion diseases, are a group of fatal neurodegenerative diseases that include chronic wasting disease (CWD) in cervids, scrapie in sheep and goats, bovine spongiform encephalopathy (mad cow disease) in cattle, and Creutzfeldt-Jakob disease and Kuru in humans. As of January 2020, CWD has been reported in free-ranging and farmed cervids in 26 states in the United States and 3 provinces in Canada (*1*). CWD-affected cervids shed infectious prions into their environment during both the preclinical and clinical stages of disease (*2*–*8*), and infectivity persists in soil (*9*–*13*), on the surface of contaminated plant leaves and roots (*14*), and in association with mineral licks (*15*). Environmental contamination with CWD prions represents a source of infectious material to which noncervid wildlife species, including raccoons and other small mammals, can be exposed.

We previously reported the transmission of the agent of CWD from white-tailed deer (*Odocoileus virginianus borealis*) and elk to raccoons through experimental intracranial inoculation (*16*). Raccoons are able to propagate CWD prions from white-tailed deer and elk but with low attack rates (25%) and with disease-associated prion protein distribution restricted to the brain (*16*).

Successful transmission of the agent of CWD from white-tailed deer to 4 species of native North America rodents has been reported previously, and meadow voles (*Microtus pennsylvanicus*) were found to be the most susceptible species (*17*). Meadow voles are known to opportunistically scavenge carcasses and engage in cannibalistic behavior (*18*), providing a plausible route for exposure to CWD and the possibility of continued disease transmission. Small rodents are a food source for predators and scavengers, including raccoons, and meadow voles and raccoons inhabit overlapping geographic ranges that also overlap with locations undergoing cervid CWD epidemics (Figure 1). Therefore, the potential for direct exposure of meadow voles and raccoons to CWD infectivity in the environment exists. Indeed, studies in Wisconsin have shown that raccoons are present at deer carcasses and gut piles with a high frequency (*19*). In addition, because raccoons are mesopredators and scavengers, there is the potential for secondary exposure of raccoons through consumption of contaminated rodents.

**Figure 1:**
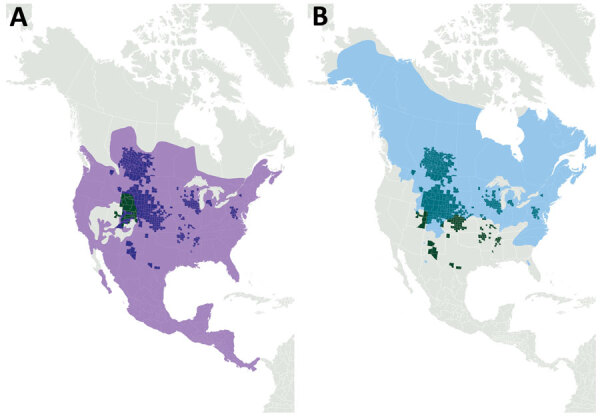
Overlap of raccoon and meadow vole distributions and chronic wasting disease epidemics, North America. A) Light purple shading indicates raccoon distribution; B) light teal shading indicates meadow vole distribution. Dark green areas and dark purple (A) and teal (B) overlays show known locations of chronic wasting disease in free-ranging cervids (as of March 2020).

To examine the potential for noncervid species to support CWD transmission, we intracranially inoculated raccoons with the agent of CWD from a white-tailed deer or with derivatives of the same inoculum after it had been passaged through meadow voles 1 or 5 times. In this study, we report the successful transmission of the agent of CWD from a white-tailed deer and vole-passaged CWD to raccoons through experimental intracranial inoculation. Our findings suggest passage of the CWD agent through voles results in a CWD agent with altered phenotypic properties.

## Materials and Methods

We sourced 17 raccoon kits (8 weeks of age) that had no previous history of prion disease from a commercial breeder and challenged them by intracranial inoculation using 0.1 mL of a 10% brain homogenate (*20*). Brain material from 3 CWD-affected donor animals generated in a previous study (*17*) were used as inocula: 1 hunter-harvested (year of harvest 2001) CWD-positive white-tailed deer that was heterozygous for glycine and serine at codon 96 of the prion protein (GS96) (CWD^Wtd^), 1 meadow vole that had been inoculated intracranially with the CWD^Wtd^ inoculum (first passage, CWD^Vole-P1^), and 1 meadow vole that had been inoculated intracranially with brain material from a fourth passage vole (fifth passage, CWD^Vole-P5^) (Table; Appendix). We inoculated raccoons in the negative control groups with brain material from a vole that had been intracranially inoculated with obex tissue from a CWD-negative deer (CWD^Neg^) (Table; Appendix). We prepared each inoculum from a single donor animal; no pooling was performed. We monitored raccoons daily and euthanized them when they showed unequivocal signs of prion disease (such as ataxia, inability to climb, or recumbency), when intercurrent illness or injury was present that could not be remedied by veterinary care, or at the end of the experiment at 35 months after inoculation. At raccoon death, we performed a full necropsy on all raccoons. We fixed 1 set of tissue samples in 10% buffered formalin, embedded in paraffin wax, and sectioned at 5 μm for microscopy examination after hematoxylin and eosin staining or immunohistochemical staining for detection of disease-associated prion protein (PrP^Sc^) by using a cocktail containing 2 monoclonal antibodies, F89/160.1.5 and F99/97.6.1 (Appendix). We froze the second set of tissues, comprising subsamples of all tissues collected into formalin, and examined selected samples for the presence of disease-associated prion protein (PrP^Sc^) by using a commercially available antigen-capture enzyme immunoassay or in-house Western blotting (Appendix).

**Table:** Summary of results of experimental inoculation of raccoons with the agent of CWD from white-tailed deer or vole-passaged CWD isolates*

Raccoon no.	Inoculum	Incubation time, mpi	Clinical signs	EIA OD	Spongiform change	Immunohistochemistry				
Brain	Retina	Pituitary	ENS	LRS
1	WTD CWD (CWD^WTD^)	21	+	4.000	+	+	+	+	–	–
2		22	+	4.000	+	+	+	–	–	–
3		27	+	3.244	+	+	+	+	+	–
4		32	–	0.095	–	–	–	–	–	–
5	1st passage CWD^WTD^ in vole (CWD^Vole-P1^)	13	–	4.000	+	+	–	NA	–	–
6		18	–	4.000	+	+	+/−	–	–	–
7		22	–	4.000	+	+	+	–	–	–
8		22	+	4.000	+	+	+	+	–	–
9		24	+	4.000	+	+	+	+	–	–
10	5th passage CWD^WTD^ in vole (CWD^Vole-P5^)	3	–	0.093	–	–	–	–	–	NA
11		17	–	4.000	+	+	+	NA	–	–
12		18	+	4.000	+	+	+	+	–	–
13		21	+	4.000	+	+	+	+	–	–
14		21	+	4.000	+	+	+	+	–	–
15	CWD-negative WTD in vole (CWD^Neg^)	35	–	0.097	–	–	–	–	–	–
16		35	–	0.095	–	–	–	–	–	–
17		35	–	0.090	–	–	–	–	–	–
18		35	–	0.109	–	–	–	–	–	–

*CWD, chronic wasting disease; EIA, antigen-capture enzyme immunoassay ; ENS, enteric nervous system; LRS, lymphoid tissues; mpi, months postinoculation; NA, not available; OD, optical density; WTD, white-tailed deer.

### Ethics Statement

This experiment was carried out in accordance with the Guide for the Care and Use of Laboratory Animals (Institute of Laboratory Animal Resources, National Academy of Sciences, Washington, DC, USA) and the Guide for the Care and Use of Agricultural Animals in Research and Teaching (Federation of Animal Science Societies, Champaign, IL, USA). The Institutional Animal Care and Use Committee at the National Animal Disease Center reviewed and approved the animal use protocols (approval no. ARS-2778).

## Results

**Figure 2:**
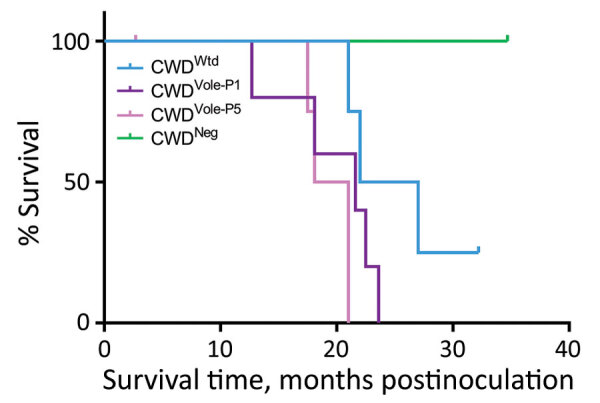
Survival curves for raccoons inoculated intracranially with the agent of CWD from white-tailed deer or vole-passaged CWD. CWD, chronic wasting disease; CWD^Neg^, CWD negative white-tailed deer; CWD^Vole-P1^, first passage (white-tailed deer to vole); CWD^Vole-P5^, fifth passage (vole to vole); CWD^Wtd^, CWD from white-tailed deer.

In the CWD^Wtd^ group, 3/4 raccoons demonstrated clinical signs consistent with prion disease (ataxia, inability to climb, recumbency); the average survival time was 23 months postinoculation (mpi) (Table). The remaining raccoon was euthanized at 32 mpi because of bilateral eye lesions; PrP^Sc^ was not detected in any tissues examined. We detected PrP^Sc^ in all raccoons in the CWD^Vole-P1^ group. Two raccoons were euthanized or found dead because of urinary tract disease, and 1 was euthanized at 22 mpi because of lameness that was not responsive to treatment. The remaining 2 raccoons exhibited ataxia and inability to climb and were euthanized at 22 and 24 mpi (Table). In the CWD^Vole-P5^ group, 2 raccoons were euthanized because of urinary tract disease at 3 mpi (PrP^Sc^ not detected) and 17 mpi (PrP^Sc^-positive). During 18–21 mpi, the remaining 3 raccoons demonstrated ataxia and inability to climb; 2 of these animals also showed head tremors (Table). All 4 raccoons in the CWD^Neg^ control group were clinically normal when they were euthanized at the end of the study at 35 mpi (Figure 2). By using antigen-capture enzyme immunoassay, we detected PrP^Sc^ in the brains of 3/4 raccoons in the CWD^Wtd^ group, 5/5 raccoons in the CWD^Vole-P1^ group, 4/4 raccoons in the CWD^Vole-P5^ group (not including the raccoon that was euthanized because of urinary tract disease at 3 mpi), and 0/4 raccoons in the CWD^Neg^ group (Table).

**Figure 3:**
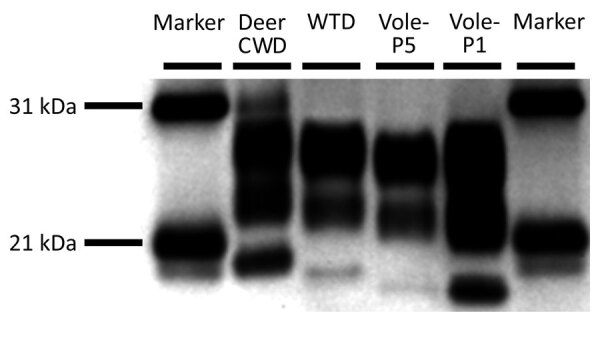
Western blots from naturally affected deer and experimentally inoculated raccoons with CWD. Lanes, from left: molecular marker; deer CWD, white-tailed deer naturally affected with CWD; WTD, raccoon 2 inoculated with brain material from the deer CWD donor animal (CWD^Wtd^); vole-P5, raccoon 14 inoculated with CWD^Vole-P5^ (fifth passage vole to vole); vole-P1, raccoon 7 inoculated with CWD^Vole-P1^ (first passage, white-tailed deer to vole); lane 6, molecular marker. CWD, chronic wasting disease.

When we analyzed brain samples by Western blot by using monoclonal PrP antibody P4, migration patterns for all animals within a treatment group were similar to each other and to the original inoculum (data not shown). When we compared samples across groups, migration patterns for vole-passaged groups were similar to each other with the unglycosylated band at ≈19 kDa. The unglycosylated band of the sample from the CWD^Wtd^ group migrated slightly higher, at 20 kDa, and that of the original donor white-tailed deer migrated slightly higher again, at ≈21 kDa (Figure 3).

**Figure 4:**
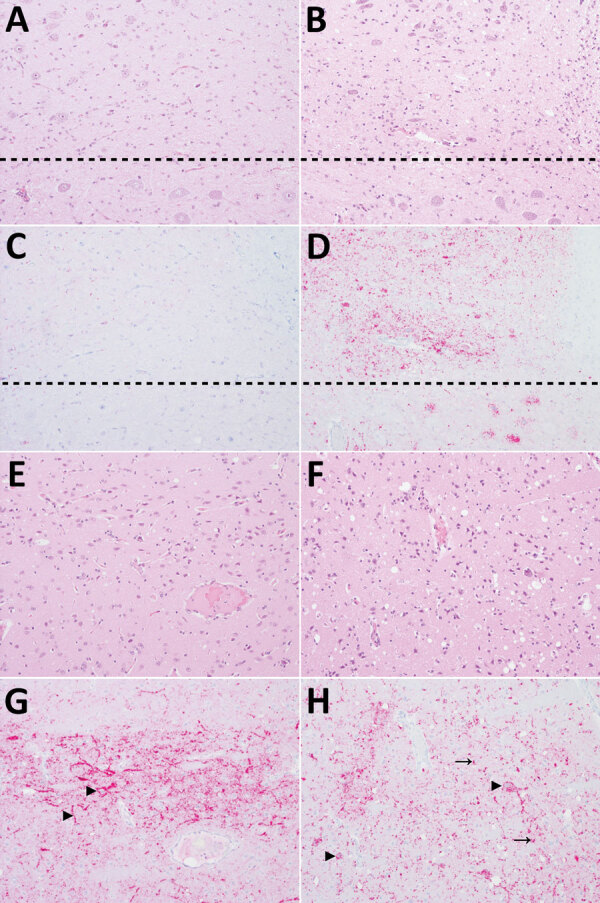
Patterns of histopathology and immunohistopathology in brains from 2 raccoons inoculated with the agent of chronic wasting disease (CWD). Panels A, C, E, and G show results for raccoon 2, inoculated with the agent of CWD from white-tailed deer; panels B, D, F, and H) show results for raccoon 9, inoculated with CWD from a vole that had been inoculated with the 4th vole-passage of the agent of CWD from white-tailed deer. All images original magnification ×20. A–D) Medulla at the level of the obex. A) Raccoon 2 shows no spongiform change in the dorsal motor nucleus of the vagus nerve (DMNV) (above dashed line) or hypoglossal nucleus (below dashed line). Hematoxylin and eosin (H&E) stain. B) Raccoon 9 shows mild to moderate spongiform change in the DMNV. H&E stain. C) Raccoon 2 shows very mild PrP^Sc^ immunoreactivity in the DMNV and no immunoreactivity in neurons of the hypoglossal nucleus. PrP antibodies F89/160.1.5 and F99/97.6.1. D) Raccoon 9 shows moderate PrP^Sc^ immunoreactivity in the neuropil of the DMNV and marked intraneuronal immunoreactivity in the hypoglossal nucleus. PrP antibodies F89/160.1.5 and F99/97.6.1. E–H) Caudate nucleus. E) Raccoon 2 shows moderate diffuse spongiform change. H&E stain. F) Raccoon 9 shows marked diffuse spongiform change. H&E stain. G) Raccoon 2 shows diffuse neuropil PrP^Sc^ immunoreactivity and prominent extracellular PrP^Sc^ accumulation on neurons (arrowheads). PrP antibodies F89/160.1.5 and F99/97.6.1. H) Raccoon 9 shows marked intracellular PrP^Sc^ immunoreactivity in neurons (arrowheads) and glial cells (arrows). PrP antibodies F89/160.1.5 and F99/97.6.1.

We examined hematoxylin and eosin–stained sections to assess pathologic changes in the brain (Figure 4). Immunohistochemical staining for PrP^Sc^ was applied to the brain and peripheral tissues to investigate the distribution of PrP^Sc^ throughout the body (Figure 4). In raccoons in the CWD^Wtd^ group, spongiform change of the neurophil was mild caudally (medulla at the level of the obex and midbrain) and moderate rostrally (thalamus and basal nuclei). Spongiform change was not observed in the dorsal motor nucleus of the vagus nerve (Figure 4, panel A) or cerebellum and was mild to moderate in the basal nuclei (Figure 4, panel E) and neocortex. In contrast, spongiform change in the vole-passaged CWD groups was moderate to marked throughout the brain, including in the dorsal motor nucleus of the vagus nerve (Figure 4, panel B), basal nuclei (Figure 4, panel F) and neocortex, and mild in the cerebellum. Intraneuronal vacuolation was only observed in 2 raccoons, both of which were from the vole-passaged CWD groups. A single intraneuronal vacuole was seen in the red nucleus of raccoon 6 (CWD^Vole-P1^) and the dorsal motor nucleus of raccoon 14 (CWD^Vole-P5^).

We detected immunoreactivity for PrP^Sc^ in the brain, spinal cord, retina, optic nerve, and/or pituitary in >2 raccoons per group (Table). We did not detect PrP^Sc^ in any lymphoid tissues sampled but was observed in the enteric nervous system of the stomach, jejunum, ileum and colon of raccoon 3 (CWD^Wtd^).

In the brains of raccoons in the CWD^Wtd^ group, the overall amount of PrP^Sc^ immunoreactivity was less in the caudal parts of the brain (Figure 4, panel C) and greater in the rostral parts of the brain (thalamus and basal nuclei) (Figure 4, panel G). Extracellular PrP^Sc^ accumulation in the neuropil and on neurons was more prominent than intraneuronal accumulation (Figure 4, panels C, G). In contrast, the pattern of PrP^Sc^ immunoreactivity was similar in raccoons in the vole-passaged CWD groups and characterized by PrP^Sc^ immunoreactivity throughout the brain with intracellular PrP^Sc^ accumulation in microglia, astrocytes, and neurons (Figure 4, panels D, H).

**Figure 5:**
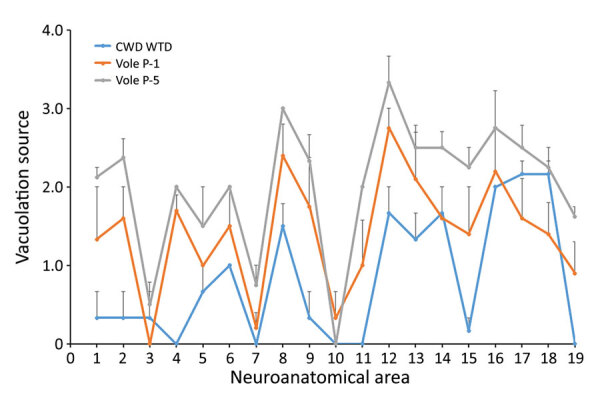
Vacuolation lesion profiles for study raccoons inoculated with the agent of CWD from WTD (blue) or inoculum prepared from the first-passage (orange) or fifth-passage (gray) of CWD WTD in voles. Error bars represent SE of the mean. CWD, chronic wasting disease; WTD, white-tailed deer.

To enable objective comparisons of the distribution and severity of spongiform change between inoculation groups, we scored the severity of vacuolation on a scale of 0–4 for 17 neuroanatomical areas and used the score to generate vacuolation lesion profiles as described previously (*21*). We made modifications to include the red nucleus and dorsal motor nucleus of the vagus nerve, which resulted in a total of 19 neuroanatomical areas examined. The distribution of vacuolation in raccoons was similar in the vole-passaged CWD groups, although the overall severity of vacuolation was greater in the CWD^Vole-P5^ group than the CWD^Vole-P1^ group (Figure 5). The pattern of vacuolation observed in raccoons in the CWD^Wtd^ group was different from raccoons in the vole-passaged groups. A trend for less severe vacuolation overall was particularly noticeable in the medulla (Figure 5, neuroanatomical areas 1–4), midbrain (Figure 5, areas 8–9), frontal cortex (Figure 5, area 13), and claustrum (Figure 5, area 17) (Appendix). 

## Discussion

We demonstrated that clinical disease developed in raccoons inoculated intracranially with the agent of CWD from white-tailed deer (CWD^Wtd^); the average incubation period was ≈23 mpi. Passage of the CWD^Wtd^ isolate through meadow voles before inoculation of raccoons with this vole-passaged CWD resulted in slightly shorter incubation periods (≈20 mpi) and different neuropathology and western blot migration pattern as compared with the original CWD^Wtd^ isolate.

We previously reported that experimental intracerebral inoculation of raccoons with an inoculum prepared from pooled brainstems from 11 CWD-affected white-tailed deer (CWD^Wtd-pool^) resulted in disease in 1/4 raccoons with an incubation period of 73 mpi and restricted distribution of PrP^Sc^ accumulation in the brain (*16*). The low attack rate and prolonged incubation period produced by the CWD^Wtd-pool^ inoculum compared with the CWD^Wtd^ inoculum reported in this study could be due to differences in the titer of PrP^Sc^ in the donor inocula. However, we consider this scenario unlikely, because all donor deer used to prepare the CWD^Wtd-pool^ inoculum and the single donor deer used to prepare the CWD^Wtd^ inoculum were positive by immunohistochemistry for PrP^Sc^. Another point of difference in disease expression produced by the CWD^Wtd-pool^ compared with the CWD^Wtd^ inoculum is the pattern of neuropathology observed in the brain: vacuolation of neuronal perikarya was widespread in the brain of the raccoon inoculated with the CWD^Wtd-pool^ (*16*) but was not observed in raccoons inoculated with CWD^Wtd^. The differences in biologic behavior of these 2 CWD isolates are most likely associated with differences in the prion protein (*PRNP*) genotype of the donor deer. Four *PRNP* polymorphisms exist in white-tailed deer: Q95H, G96S, A116G, and Q226K (reviewed in S.J. Robinson et al. [*22*]). At codon 96, the S96 allele is associated with reduced CWD prevalence (*23*–*26*) and prolonged incubation periods (*27*). Donor deer for the CWD^Wtd-pool^ inoculum were all GG96 *PRNP* genotype (*16*), whereas the donor deer for the CWD^Wtd^ inoculum was GS96 *PRNP* genotype. We did not expect that inoculum containing the S96 allele would produce disease in raccoons more efficiently than inoculum exclusively containing the G96 allele. In addition, sequencing of *PRNP* from raccoons in a previous study showed that raccoons are homozygous for glycine at codon 96 (GG96) (S.J. Moore, unpub. data). Therefore, our results suggest that patterns of disease susceptibility associated with *PRNP* polymorphisms at codon 96 in CWD-affected white-tailed deer might not be a useful predictor of disease outcomes in intracranially inoculated raccoons. Further studies are under way to investigate the biologic behavior in raccoons of CWD from a single-source GG96 white-tailed deer.

Cross-species transmission of CWD isolates might result in a change in the biochemical properties of the disease-associated prion protein or the biologic behavior of the prion strain, such as adaptation to its host, or both (*16*,*28*–*33*). The pattern of PrP^Sc^ accumulation in the brain of the raccoon inoculated with CWD^Wtd-pool^ in our previous study (*16*) was similar to raccoons inoculated with the CWD^Wtd^ inoculum in this study and was characterized by prominent linear and perineuronal PrP^Sc^ accumulation, although this comparison is limited by the small number of animals available for examination.

Both vole-passaged CWD isolates produced similar disease phenotypes in raccoons with regards to incubation periods, western blot migration patterns, and neuropathology. The patterns of spongiform change and PrP^Sc^ accumulation in the brains of raccoons inoculated with vole-passaged CWD isolates were similar to each other and different from those observed in the brains of raccoons inoculated with the CWD^Wtd^ isolate. Inoculum-associated differences in western blot migration patterns were observed (i.e., similar patterns for vole-passaged CWD isolates and a different pattern for the CWD^Wtd^ isolate). In addition, the migration pattern of the original CWD-affected white-tailed deer donor (Figure 3, deer CWD, unglycosylated band at ≈21 kDa) was different from both the raccoon-passaged CWD^Wtd^ isolate (Figure 3, white-tailed deer, 20 kDa) and the vole-passaged CWD isolates (Figure 3, vole-P1 and vole-P5, 19 kDa) after passage through raccoons. Therefore, passage of CWD^Wtd^ through voles appears to result in a change in the biologic behavior of this prion isolate when inoculated intracranially into raccoons. This finding raises the possibility for emergence of novel CWD strains after passage in off-target species through host-driven selection of a strain present in the donor inoculum (*29*,*34*). The original inoculum was derived from a white-tailed deer with the GS96 *PRNP* genotype, and propogation of CWD prions on S96 PrP^C^ results in the formation of alternative PrP^Sc^ conformers (*34*). We are unsure what role the genotype of the deer in the inoculum might have played in the change in biologic behavior noted after passage through voles. Because intracranial inoculation is not a natural route for exposure of raccoons to CWD infection, oral transmission studies are underway to characterize the biologic behavior of the CWD^Wtd^ and vole-passaged CWD isolates using a more natural route of exposure.

We observed a single intraneuronal vacuole in the red nucleus of raccoon 6 (CWD^Vole-P1^ group) and the dorsal motor nucleus of raccoon 14 (CWD^Vole-P5^ group). Intraneuronal vacuolation was previously reported as an incidental finding in the brainstem (including facial and pontine nuclei), cerebellar roof nuclei, and cerebrum of raccoons (*35*,*36*). In those raccoons, no evidence of concurrent neuropil vacuolation, neuronal degeneration, or astrocytosis was seen. In contrast, widespread neuropil vacuolation throughout the brains of raccoons 6 and 14, and strong PrP^Sc^ immunoreactivity in vacuolated neurons was evident; therefore, the intraneuronal vacuoles observed in these raccoons are likely associated with prion infection.

Although PrP^Sc^ immunoreactivity was widely distributed throughout the brain and spinal cord, we did not generally observe involvement of the peripheral nervous system, with the exception of 1 raccoon (3) inoculated with CWD^Wtd^, in which PrP^Sc^ immunoreactivity was present in the enteric plexi of the stomach, jejunum, ileum, and colon. The general lack of peripheral nervous system involvement is likely because raccoons were inoculated through the intracranial route that bypasses centripetal spread of PrP^Sc^ from the alimentary tract to the brain along parasympathetic nerves, as is observed in orally infected deer (*37*). Instead, PrP^Sc^ immunoreactivity in the enteric nervous system of raccoon 3 was likely the result of centrifugal spread from the central nervous system. Why PrP^Sc^ immunoreactivity was observed throughout the spinal cord in all raccoons is unclear, but enteric nervous system involvement was only seen in raccoon 3. Raccoon 3 was the longest surviving raccoon (27 mpi), so the possibility exists that, had other raccoons not succumbed to clinical disease, there might have been time for transport of PrP^Sc^ to the enteric nervous system. Inoculation of raccoons by the oral route is needed to improve our understanding of the pathogenesis of CWD in raccoons when exposure occurs by a more natural route.

The longest surviving CWD-inoculated raccoon (4) was euthanized at 32 mpi because of bilateral eye lesions. Histopathologic examination resulted in a diagnosis of multicentric lymphoma, and PrP^Sc^ was not detected in any tissues. Clinical disease and widespread PrP^Sc^ accumulation at 21–27 mpi developed in all other raccoons in the CWD^Wtd^ group (n = 3). The reason for the unexpected negative result for raccoon 4 is unclear but could include experimental error or host factors. With regard to experimental inoculation, all inocula were prepared and all raccoons were inoculated on the same day, so the likelihood is very low that this raccoon did not receive the correct inoculum. The strongest determinant of susceptibility to prion diseases is the host *PRNP* sequence. No unique single nucleotide polymorphisms were detected in the *PRNP* open reading frame of raccoon 4 (S.J. Moore, unpub. data). It is tempting to speculate that host, genetic, or immunological factors outside of the *PRNP* open reading frame that contributed to the development of neoplasia might have had a suppressive effect on PrP^Sc^ accumulation.

Prion diseases of free-ranging animals do not exist in isolation. Meadow voles and raccoons are widespread in North America, and their habitat ranges overlap with those of CWD-affected white-tailed deer and other cervids. Therefore, a substantial potential for exposure of these or other off-target species to CWD infectivity in the environment exists. We have demonstrated that CWD^Wtd^ from a GS96 white-tailed deer transmitted readily to raccoons. Passage of this isolate through voles followed by intracranial inoculation of raccoons with vole-derived inoculum resulted in disease with different biologic characteristics and neuropathology than the original CWD^Wtd^ isolate. These results provide strong evidence for the emergence of a novel strain of CWD after passage in meadow voles and raccoons. Therefore, interspecies transmission of CWD prions between cervids and noncervid species that share the same habitat might represent a confounding factor in CWD-management programs. In addition, passage of CWD prions through off-target species might represent a source of novel CWD strains with unknown biologic characteristics, including zoonotic potential. Characterization of the biologic behavior of CWD isolates after cross-species transmission will help us develop more effective management strategies for CWD-affected populations.

Appendix Additional information about attack rates and incubation periods for chronic wasting disease in raccoons after passage through meadow voles.
