# Supplementary material for: Increased Attack Rates and Decreased Incubation Periods in Raccoons with Chronic Wasting Disease Passaged through Meadow Voles
Source: Emerg Infect Dis. 2022 Apr;28(4):793–801. doi: 10.3201/eid2804.210271 (PMC8962881; doi:10.3201/eid2804.210271)
Supplement: Appendix — Additional information about attack rates and incubation periods for chronic wasting disease in raccoons after passage through meadow voles. [file 21-0271-Techapp-s1.pdf]

Article DOI: https://doi.org/10.3201/eid2804.210271

Increased Attack Rates and Decreased Incubation Periods of Chronic Wasting Disease in Raccoons after Passage through Meadow Voles

Appendix

Materials and Methods

Inoculum

The CWD^{Wtd} inoculum (10% [wt/vol] obex tissue in phosphate-buffered saline) was prepared from a single hunter-harvested (year of harvest 2001) CWD-positive white-tailed deer (*Odocoileus virginianus*) from southern Wisconsin that was heterozygous for glycine and serine at codon 96 of the prion protein (GS96) as described previously (*1*). The vole-passaged CWD inocula (10% [wt/vol] whole brain in phosphate-buffered saline) were prepared from individual meadow voles (*Microtus pennsylvanicus*) as described previously (*1*). Donor voles had been inoculated intracranially with either the CWD^{Wtd} inoculum (first passage, $CWD^{Vole\text{-}P1}$), brain homogenate from a fourth passage vole (fifth passage, $CWD^{Vole\text{-}P5}$), or brain homogenate from a vole inoculated with homogenate from the obex tissue of a CWD-negative deer (CWD^{Neg}). Each inoculum was prepared from a single vole source; no pooling was performed.

Animals

Eight-week-old raccoon kits were sourced from a commercial breeder that had never had a reported case of prion disease (*2*). The kits were divided into 4 groups: group 1 (n = 4) was inoculated with CWD^{Wtd}; group 2 (n = 5) was inoculated with $CWD^{Vole\text{-}P1}$; group 3 (n = 4) was inoculated with $CWD^{Vole\text{-}P5}$; group 4 (n = 4) was inoculated with CWD^{Neg} and served as negative controls.

Animal Housing

Raccoons were housed in a Biosafety Level 2 containment facility at the National Animal Disease Center (Ames, Iowa, USA) and monitored daily for clinical signs of prion disease. Raccoons were euthanized when they showed unequivocal signs of prion disease such as ataxia, inability to climb, or recumbency; when euthanasia was necessary due to intercurrent illness or injury that could not be remedied by veterinary care; or at the end of the experiment at 35 months after inoculation.

Sample Collection

The following samples were collected in postmortem examination: brain, spinal cord, retina, peripheral nervous tissue (ganglia: dorsal root, trigeminal; nerves: optic, sciatic), lymphoid tissues (thymus, 3rd eyelid; tonsils: pharyngeal, palatine, rectal; lymph nodes: mesenteric, popliteal, prescapular, retropharyngeal), adrenal gland, anal gland, gall bladder, gastrointestinal (esophagus, stomach, duodenum, jejunum, ileum, cecum, colon, rectum), heart, diaphragm, skeletal muscle (biceps, masseter, psoas, triceps), tongue, kidney, urinary bladder, liver, pancreas, pituitary, salivary gland, skin (lip margin, nose skin, paw skin), spleen, thyroid, turbinate, trachea, and lung.

Vacuolation Lesion Profiling

The following neuroanatomical areas were assessed for vacuolation lesion profiling: 1, nucleus of the solitary tract; 2, nucleus of the spinal tract of the trigeminal nerve; 3, hypoglossal nucleus; 4, dorsal motor nucleus of the vagus nerve; 5, vestibular nuclear complex; 6, cochlear nucleus; 7, cerebellar vermis; 8, central gray matter; 9, rostral colliculus; 10, red nucleus; 11, medial geniculate nucleus; 12, hypothalamus; 13, nucleus dorsomedialis thalami; 14, nucleus ventralis lateralis thalami; 15, frontal cortex; 16, septal nuclei; 17, caudate nucleus; 18, putamen; 19, claustrum.

Immunohistochemistry

Immunostaining for the detection of disease-associated prion protein (PrP^{Sc}) was performed as described previously (*3*) using a cocktail containing 2 monoclonal antibodies, F89/160.1.5 and F99/97.6.1, each applied at a concentration of 5 µg/mL with an automated processor.

Antigen-Capture Enzyme Immunoassay (EIA)

Frozen brainstem samples were homogenized in 1X phosphate-buffered saline at a concentration of 20% wt/vol and assayed with a commercially available antigen-captire enzyme immunoassay kit (HerdChek BSE-Scrapie Ag Test Kit; IDEXX Laboratories, https://www.idexx.com) as previously described (*4*).

Western Blotting

Samples were collected from the brainstem at the level of the obex and western blotting was performed as previously reported (*5*) after preparing the homogenate in a commercial solution (TeSeE Western Blot; BIO-RAD, https://www.bio-rad.com). Western blots were developed by using mouse anti-prion protein monoclonal antibody 6H4 (ThermoFisher Scientific, https://www.thermofisher.com) at a 1:10,000 dilution.

References

1. Heisey DM, Mickelsen NA, Schneider JR, Johnson CJ, Johnson CJ, Langenberg JA, et al. Chronic wasting disease (CWD) susceptibility of several North American rodents that are sympatric with cervid CWD epidemics. J Virol. 2010;84:210–5. PubMed https://doi.org/10.1128/JVI.00560-09

2. Hamir AN, Miller JM, Cutlip RC, Stack MJ, Chaplin MJ, Jenny AL, et al. Experimental inoculation of scrapie and chronic wasting disease agents in raccoons (*Procyon lotor*). Vet Rec. 2003;153:121–3. PubMed https://doi.org/10.1136/vr.153.4.121

3. Greenlee JJ, Smith JD, West Greenlee MH, Nicholson EM. Clinical and pathologic features of H-type bovine spongiform encephalopathy associated with E211K prion protein polymorphism [Erratum in: PLoS One. 2013;8:1]. PLoS One. 2012;7:e38678. PubMed https://doi.org/10.1371/journal.pone.0038678

4. Smith JD, Greenlee JJ. Detection of misfolded prion protein in retina samples of sheep and cattle by use of a commercially available enzyme immunoassay. Am J Vet Res. 2014;75:268–72. PubMed https://doi.org/10.2460/ajvr.75.3.268

5. Greenlee JJ, Smith JD, Kunkle RA. White-tailed deer are susceptible to the agent of sheep scrapie by intracerebral inoculation. Vet Res (Faisalabad). 2011;42:107. PubMed https://doi.org/10.1186/1297-9716-42-107
